# Characterization of the infection process by *Peronospora belbahrii* on basil by scanning electron microscopy

**DOI:** 10.1016/j.heliyon.2019.e01117

**Published:** 2019-01-31

**Authors:** Guirong Zhang, Arthur Thompson, David Schisler, Eric T. Johnson

**Affiliations:** Crop Bioprotection Research Unit, USDA ARS, Peoria, IL, 61604, USA

**Keywords:** Plant biology, Microbiology

## Abstract

Basil downy mildew caused by *Peronospora belbahrii* is a disease of sweet basil (*Ocimum basilicum*) production worldwide. In this study, sweet basil was grown in plant growth chambers and inoculated with sporangia of *P. belbahrii* harvested from previously infected plants. Plants were placed in closed, clear plastic bags and leaves harvested over time and observed using scanning electron microscopy. In most cases, sporangia germinated myceliogenically on abaxial and adaxial leaf surfaces as early as three days after inoculation. Germ tubes and the tips of hyphae ramifying on leaf surfaces directly penetrated basil leaves to initiate the infection process. Hyphal growth was not observed to gain entrance to the interior of leaves through stomata, though growth over these openings was observed. Most frequently, seven days after inoculation, one or more sporangiophores grew through stomata to produce new sporangia on both the abaxial and adaxial surfaces of leaves. Macroscopic signs of infection were visible on both sides of leaves approximately ten days after inoculation under the conditions of this study. These results contribute to a better understanding of the infection process and disease onset of *P. belbahrii* and should help in the development of more effective measures for reducing basil downy mildew.

## 1 Introduction

Basil downy mildew, caused by *Peronospora belbahrii* Thines (Belbahri et al., 2005; Thines et al., 2009), is a destructive disease of sweet basil (*Ocimum basilicum* L., Lamiaceae) (Ronco et al., 2009). In the United States, it was first reported in south Florida in October 2007 (Roberts et al., 2009) and downy mildew has been observed on basil in multiple states every year since 2008 (Blomquist et al., 2009; Wick and Brazee, 2009; McGrath et al., 2010; Mersha et al., 2013; Wyenandt et al., 2015). In Canada, the disease was first reported in 2011 (Saude et al., 2013). Globally, the disease has become a serious concern in several countries (Garibaldi et al., 2004, 2005; McLeod et al., 2006; Khateri et al., 2007; Ronco et al., 2009). The pathogen needs high relative humidity (at least 85%) or wet leaves to infect a plant, and is favored by moderate (20 °C) rather than higher temperatures (Wyenandt et al., 2015). Though some downy mildews form resistant oospores that can survive in the absence of a living host plant, reports of oospore formation by *P. belbahrii* are few (Elad et al., 2016) and do not include North America. Currently, therefore, it is unlikely that the disease will persist in field plantings from year to year in North America, at least in areas where freezing winter temperatures occur.

Since 2007 when downy mildew first was reported in the U.S., losses due to the disease were estimated to be in the tens of millions of dollars (Wyenandt et al., 2015). Losses in basil production, particularly due to the disease in the United States over the last few years, coupled with increasing consumer demand have increased the need for importing basil from other countries (Wyenandt et al., 2015). However, the discovery of downy mildew infected basil plant material at ports of entry has limited the use of this option for meeting demand. Reducing the impact of disease via fungicide application is promising for increasing production though chemical fungicides provide varying levels of control and current organic control measures are insufficient (Mersha et al., 2011; Raid, 2011a, 2011b; Allen and Saska, 2013; Babadoost and DeYoung, 2012, 2013; Gilardi et al., 2013; Patel et al., 2013; McGrath and LaMarsh, 2015). Pathogen resistance to fungicides has also been reported (Cohen et al., 2013a). Breeding for varieties of sweet basil resistant to downy mildew also is promising and has yielded highly resistant germplasm (Wyenandt et al., 2010; Djalali Farahani-Kofoet et al., 2014; Pyne et al., 2014; McGrath et al., 2014; Ben-Naim et al., 2018).

Clear documentation of the infection process by the pathogen and its subsequent development of fructifications after host colonization would greatly benefit research efforts to overcome the aforementioned obstacles to reducing the impact of downy mildew on basil. Basil downy mildew is commonly described as sporulating on the abaxial side of infected leaves (Gilardi et al., 2013; Mersha et al., 2013) with dark purplish-brown sporangia borne on sporangiophores that are produced on the underside of leaves during favorable environmental conditions for disease development. Recently, the possibility of sporulation also taking place on the adaxial side of infected leaves was raised (Cohen et al., 2017). Additional study to clarify the location of sporulation on host leaves by the pathogen is warranted.

The first symptom of host infection is a slight chlorosis (yellowing) of the adaxial surface of infected leaf tissue, usually in the mid-rib area (Mersha et al., 2013). Leaf wetness for 24 hours after symptom development results in prolific sporulation and can result in rapid spread of the disease. In the field or greenhouse, basil downy mildew can develop and spread rapidly throughout plantings during periods of high humidity, mild temperatures, poor air circulation, and extended durations of leaf wetness (Garibaldi et al., 2007; Cohen et al., 2013b). Systemic spread of the pathogen in basil plants (Djalali Farahani-Kofoet et al., 2012) has the potential to result in large areas of secondary sporulation of the pathogen on leaves even after limited points of primary infection of the host. Studies to date have not attempted to document, on a daily basis, the infection and secondary inoculum production process by *P. belbahrii* on sweet basil. In the current study we examine this process on a daily basis with scanning electron microscopy. Results will clarify the details of the infection and subsequent sporulation process of this pathogen which should be helpful in guiding the development of new resistant varieties and control measures against *P. belbahrii*.

## 2 Materials and methods

### 2.1 Inoculation of basil plants in growth chamber

Seeds of Fusarium resistant basil hybrid AROMA 2 OG F1 (*Osmium basilicum*) (Johnny's Selected Seeds, Winslow, Maine) were stored at 4 °C. Seeds were planted in six, 13 × 13 cm punnets containing 3 × 4 cells/punnet. Punnets were, in turn, contained within a 25 × 50 cm open flat tray. Three seeds per well were placed on the surface of pasteurized (60 °C air/steam for 30 min after product reached temperature) soil mix (Sunshine^®^ Propagation Mix, Sungrow Horticulture, Seba Beach, AB, Canada) supplemented with 0.5 g kg^−1^ micromax (Scotts-Sierra Horticultural Products Company, Marysville, OH) and 3.0 g kg^−1^ osmocote 15-15-15 (Everris NA Inc., Dublin, OH). Basil plants in the tray were grown in a plant growth chamber with a photoperiod of 13 h day^−1^, light intensity of 600 μEm^−2^s^−1^, relative humidity of 60–80%, and temperatures of 23 °C day and 18 °C night. Water was added to the open flat tray daily as needed.

The isolate of *P. belbahrii* used in these studies was obtained from basil plants that were collected in Danville, Illinois in 2014 and exhibited symptoms and signs of downy mildew infection. To maintain a source of infected basil seedlings from which sporangia were harvested for experimental use, sporangia from an infected basil plant of the Danville collection were washed from infected leaves. These were then used to inoculate 3–5 week old basil seedlings by spraying an aqueous suspension of sporangia at approximately 5 × 10^4^ sporangia mL^−1^ onto seedlings until runoff. Inoculated plants in trays were watered to soil saturation and placed in a 50 × 76 cm 4 mil poly bag (Uline, Chicago). The bags were sealed to create a relative humidity of 95–100% (Thermadata^TM^ temperature and humidity logger, ThermoWorks, American Fork, UT) within the bag, and the bagged trays placed in a plant growth chamber with the same growing conditions as described above. The temperatures inside the bags were 26 °C during the day and 18 °C at night. Sporangia developed after approximately 7 days and these sporangia were used to inoculate new basil plants every 10–14 days to maintain a constant source of *P. belbahrii* sporangial inoculum.

To initiate a microscopy experiment, sporangia were obtained from infected basil leaves 8 days after they were inoculated with sporangia of *P. belbahrii*. Sporangia and sporangiophores were dislodged into chilled sterile water (10–15 °C) by submerging leaves in the chilled water and dislodging sporangia by gently rubbing the leaf surface using a soft brush. The suspension of sporangia was then filtered through three layers of cheese cloth to remove mycelial fragments. Sporangia concentration was determined using a hemocytometer and adjusted to 5 × 10^4^ sporangia mL^−1^. Basil plants for experimental use were grown in a separate pathogen-free growth chamber until the first three true leaves had fully expanded (15–17 days after planting (DAP)). The leaves then were sprayed with the sporangial suspension until run-off using a handheld pump-action mister. Inoculated basil seedlings in punnets were then enclosed in plastic bags and incubated under the conditions of relative humidity, temperature and light periodicity described earlier with leaves destructively sampled periodically for microscopic examination as described below.

### 2.2 Infection characterization using scanning electron microscopy (SEM)

Samples of basil leaf tissue (at least five 0.5 × 0.5 cm sections) were taken daily for nine days after plant inoculation with sporangia (5 × 10^4^ mL^−1^). Daily samples from basil plants that were not inoculated with the pathogen were also taken in the same manner. Samples were placed into separate 1 dram vials and soaked in 2.5% glutaraldehyde (Sigma Life Science, St. Louis, MO) fixative overnight. Leaf pieces that had been inoculated with sporangia, or not, were soaked in 1X phosphate buffered saline pH7 (PBS) for 10 min followed by three 10 minute washes with dH_2_O. Specimens were post-fixed in 2% osmium tetroxide (Sigma Life Science, St. Louis, MO) for 45 min and again washed three times in distilled water.

Tissue samples then were dehydrated in a series of ethanol and water solutions (25%, 50%, 75%, 95% and three times in 100% ethanol); samples were gradually infiltrated in each solution for at least 30 min. The tissues samples suspended in the final 100% ethanol solution were held overnight. Tissue samples were then critical-point dried with a carbon dioxide critical point dryer (Tousimis Research Corp., Rockville, MD), mounted abaxial side up on aluminum stubs with conductive carbon tape (SPI Supplies, West Chester, PA), and coated for 2 min with gold in a sputter coater (Structure Probe, Inc., West Chester, PA). The SEM images were obtained on a JEOL JSM-6010LA. Typical operating conditions were accelerating voltage of 10kV and a spot size of 30. Changes in morphology of the pathogen observed in the SEM images were noted.

## 3 Results

**Fig. 1:**
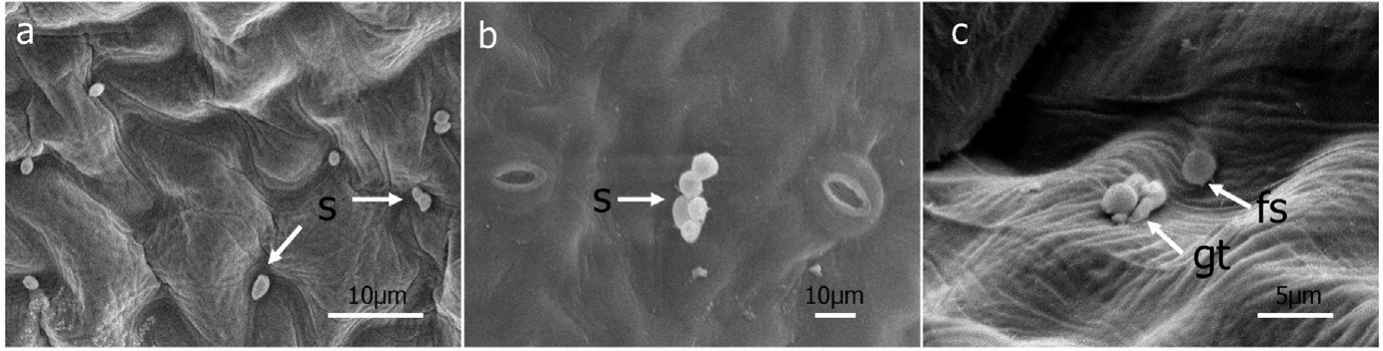
Scanning electron micrographs of sporangia of *Peronospora belbahrii* soon after inoculation onto basil leaves. a, Ungerminated sporangia (s), 1 day after inoculation (DAI), b, Ungerminated sporangia 2 DAI, c, Germinated sporangia 3 DAI with germ tube (gt) and fine structures (fs).

**Fig. 2:**
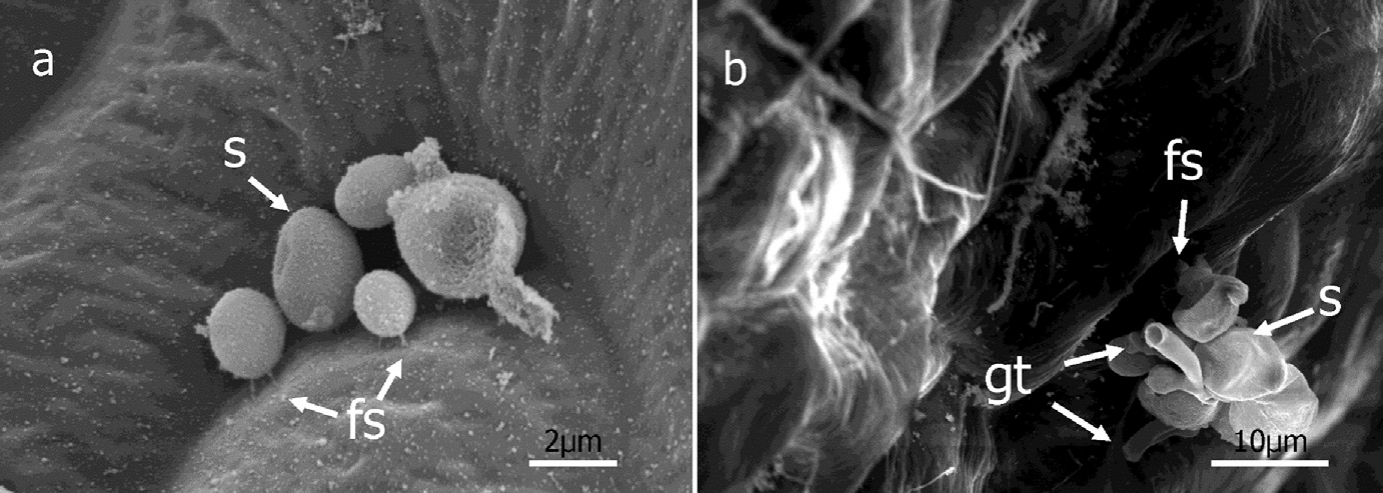
Sporangia several days after inoculation onto basil leaves. a, Ungerminated sporangia (s) with fine structure (fs) 7 DAI, b, Collapsed sporangia with germ tubes (gt) and fine structure 8 DAI.

**Fig. 3:**
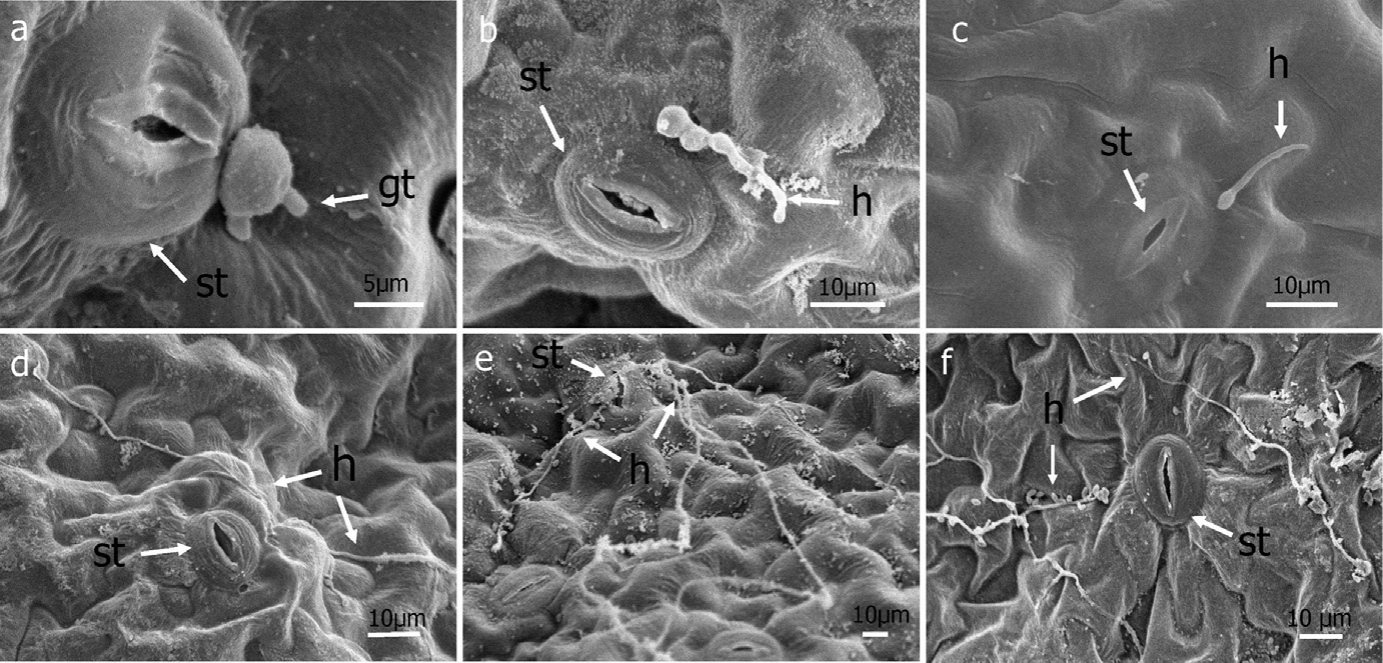
Scanning electron micrographs of germ tubes (gt) and hyphae (h) 3 DAI. a-f, Hyphal growth not oriented towards stomata (st) nor do hyphae grow into the leaf interior via entrance through stomata.

Two days after inoculation (DAI), sporangia commonly showed the first sign of swelling on the apical end. Sporangia frequently germinated myceliogenically (Figs. 1, 2, and 3) from their apical end but occasionally from both their apical and basal ends. The majority of sporangia germinated by 3 DAI, though additional sporangia germinated as late as 6 DAI. Some sporangia had fine structures that connected sporangia to leaves starting at 3 DAI (Fig. 1). Examples of this occurred throughout 9 DAI (Fig. 2) but the majority of sporangia with fine structures that appeared attached to the leaf surface did not germinate via a germ tube. In some instances, sporangia showing fine structures germinated but in most cases both the germ tubes and the sporangia subsequently collapsed.

**Fig. 4:**
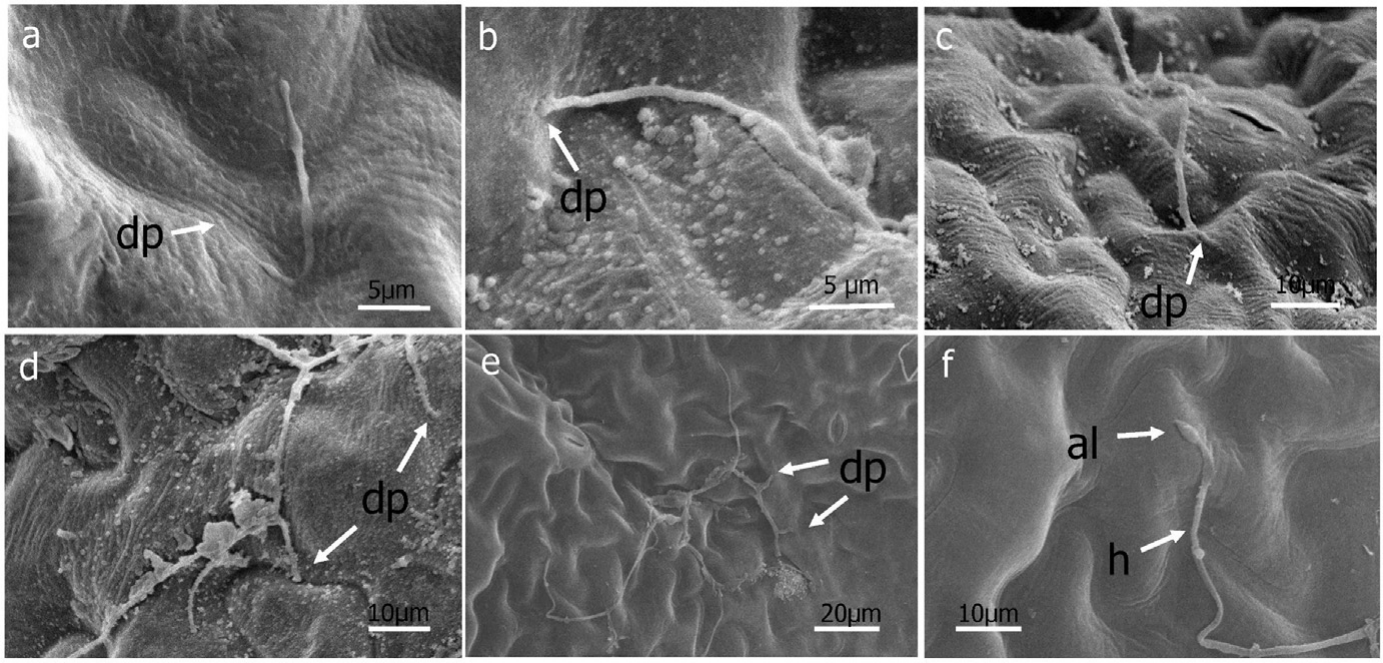
a–e, Direct penetration (dp, arrow) of basil leaves at terminus of hyphal growth, f, Appressorium-like structure (al) at end of a hypha (h).

**Fig. 5:**
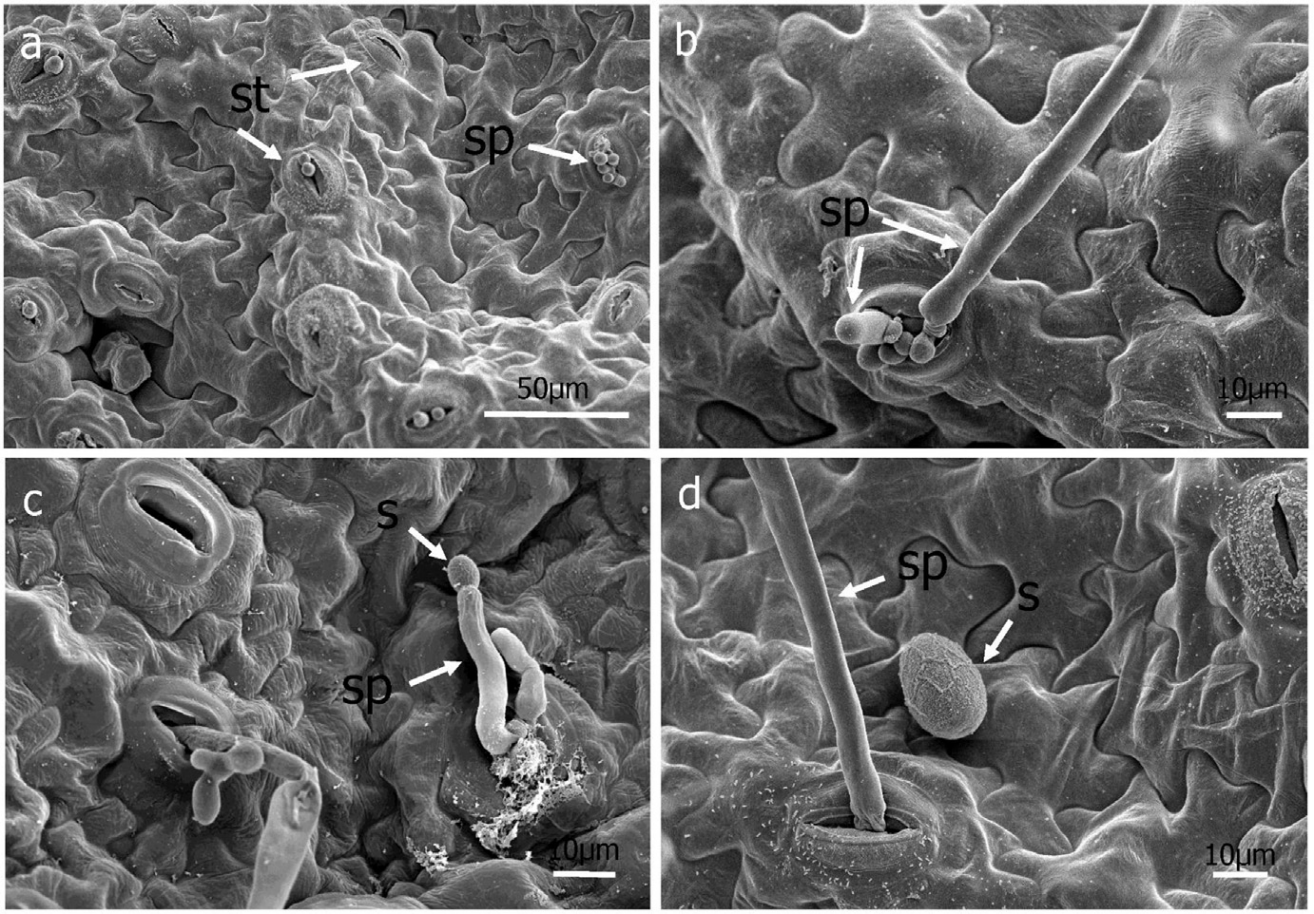
Sporangiophores on the surface of basil leaves. a, Sporangiophores (sp, arrow) emerging through stomata (st) 6 DAI, b, Old and new sporangiophores exiting from the same stoma 9 DAI, c, Sporangia (s) produced from newly formed sporangiophore 14 DAI, d, Secondary inoculum in form of a sporangium produced from a sporangiophore 14 DAI.

**Fig. 6:**
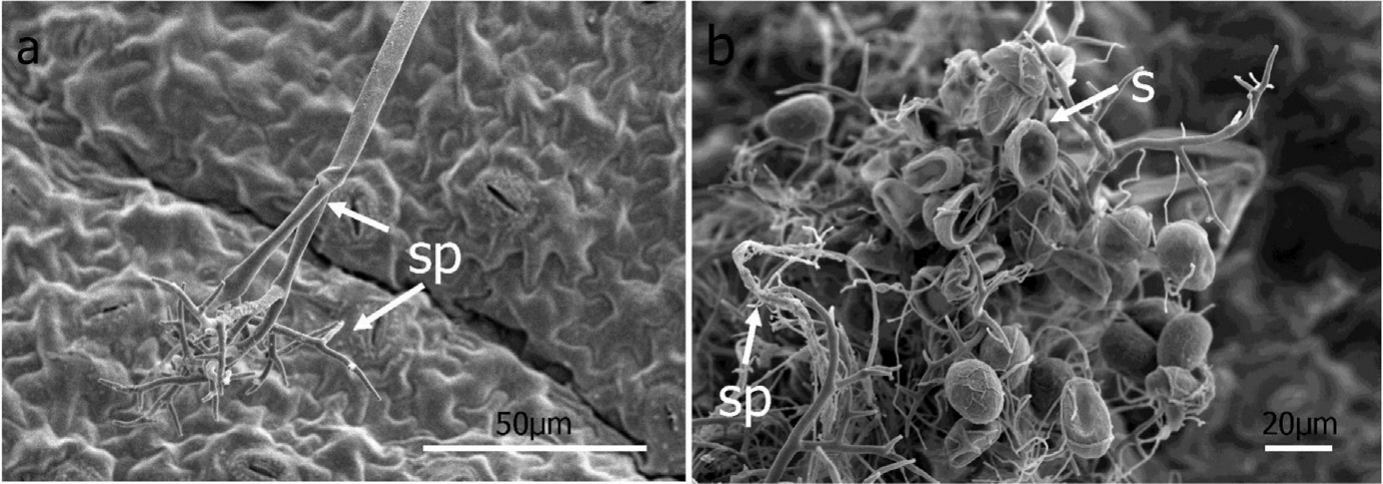
a, SEM micrograph of sporangiophore (sp) of *P. belbahrii* 14 DAI showing a typical dichotomous branching pattern and bearing sporangia at the terminus of individual sporangiophore branches, b, collapsed sporangiophore and sporangia (s) of *P. belbahrii*.

**Fig. 7:**
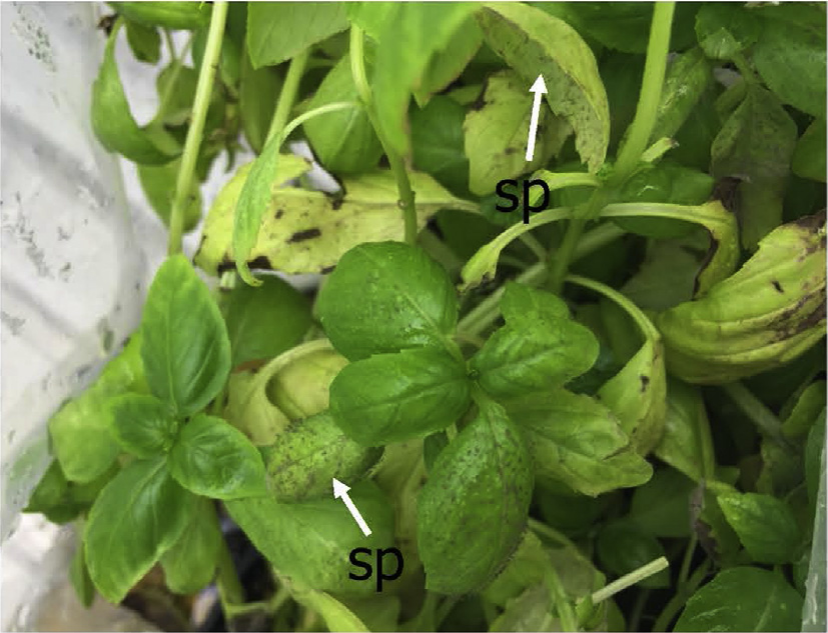
Sporangiophores (sp) of *P. belbahrii* appear on both the abaxial and adaxial surfaces of leaves on a basil plant.

Under the conditions of this study, germ tubes from sporangia grew in multiple directions and did not grow pr eferentially towards stomata (Fig. 3). When mycelia did happen to pass over a stoma, no instances of ingress into the leaf via the stomatal opening were observed (Fig. 3). Hyphae were observed to penetrate directly through the abaxial (Fig. 4) and adaxial (data not shown) surfaces of basil leaves starting at 3 DAI. Appressorium-like structures were rarely seen at the site of leaf penetration at the terminus of a hypha of the pathogen (Fig. 4). As early as 6 DAI but more commonly at 7 DAI, hyphae were observed that grew from the interior of infected basil leaves to the leaf exterior via stomata to form sporangiophores (Fig. 5). Sporangiphores first emerged via stomata on the abaxial surface of leaves but then also from stomata on the adaxial surface of leaves 1–2 days later. Also during this time, additional hyphae emerged from the leaf interior to form additional sporangiophores, in some cases many from the same stoma (Fig. 5). Sporangia were observed on recently formed and initially formed sporangiophores at every DAI from day 6 through day 14 (Figs. 5 and 6), with some sporangiophores bearing sporangia as soon as they grew out from stomata (Fig. 5). The sporangiophores of *P. belbahrii* branched at acute angles with pointed tips which bore sporangia. The sporangiophores branched irregularly, and later, dichotomously (Fig. 6). Sporangia that were apparently produced from infections that resulted from the initial inoculation of plants had in some cases germinated by approximately 10 DAI. Sporangiophores tended to collapse after shedding sporangia (Fig. 6). Some sporangia that remained attached to sporangiophores during this process also collapsed (Fig. 6). On young plants, sporangiophores that bore sporangia were visible to the unaided eye on both the abaxial and adaxial surfaces of basil leaves at 10 DAI (Fig. 7).

## 4 Discussion

After contact with a plant, pathogenic fungi and oomycetes gain entrance to internal plant tissues of the host via indirect or direct penetration (Moore-Landecker, 1990). Direct penetration through an intact epidermis can occur after the formation of an infection structure such as an appressorium while indirect penetration may occur via a pathogen entering a plant through a natural plant opening such as a stoma (Pritsch et al., 2000). The method of host penetration employed by a pathogen can depend on environmental factors (Howard, 1997). Under the environmental conditions of this study, sporangia of *P. belbahrii* germinated in three to five days by either one or two germ tubes. Thines et al. (2009) documented germination of a sporangium of *P. belbahrii* using a photomicrograph that showed two germ tubes emerging from a single sporangiospore. Koroch et al. (2013), however, reported germination of sporangia of this species via one germ-tube. Both myceliogenic germination possibilities were seen under the conditions of the current study. Under the experimental conditions used by Cohen and Ben-Naim (2016), a mefenoxam-resistant isolate of *P. belbahrii* from Israel initiated infection with as little as 4 h of free leaf moisture. Differing experimental conditions, isolates and climatological adaptation of those isolates could explain the observed differences seen regarding the timing of infection. Studies comparing the infection process of a variety of *P. belbahrii* isolates under standardized conditions are needed.

Germ tubes rarely formed appressorium-like structures prior to penetrating the leaf epidermis (Fig. 4). Usually, direct penetration appeared to take place without differentiation of a hypha at the point of penetrating the leaf surface (Fig. 4). Penetration of the epidermis of basil leaves by germ tubes growing through stomata was not observed in this study after viewing more than three-hundred germinated sporangia using scanning microscopy. Hyphae were not observed to grow preferentially towards stomata (Fig. 3). Hyphae occasionally grew directly over stomata but without ingress to the leaf interior (Fig. 3). This result differs from a previous study (Koroch et al., 2013) that reported germ tubes from sporangia of *P. belbahrii* entered basil leaves through stomata but agrees with a report of direct penetration of the epidermis of basil (Cohen and Ben-Naim, 2016). Basil leaf penetration by *P. belbahrii* via appressoria and stomatal openings has recently been reported (Cohen et al., 2017). Pre-entry, entry and colonization of a host by a pathogen can be influenced by environmental factors including temperature, leaf wetness and light (Cohen et al., 1971, 2013b; Fried and Stuteville, 1977; Garibaldi et al., 2007). Because each of these factors in our study were not identical to those reported by Koroch et al. (2013) or Cohen et al. (2017), additional studies will be needed to ascertain if environmental conditions dictate the mode of host penetration utilized by *P. belbahrii* and, concomitantly, to determine if reduced stomatal number is a phenotype to pursue in breeding for basil lines resistant to downy mildew (Homa et al., 2016).

Some plant pathogens form specialized infection structures (e,g. appressoria and infection cushions) such as the wheat stem rust fungus (*Puccinia graminis tritici*) (Leonard and Szabo, 2005), and *Rhizoctonia solani* (Armentrout and Downer, 1987; Basu et al., 2016), respectively. Alternatively, encysted zoospores of the oomycete *Phytophthora cinnamomi* can rapidly attach to a host surface and directly penetrate without apparent production of specialized infective structures (Redondo et al., 2015). In this study, fine structures were observed connecting ungerminated sporangia to the leaf surface as early as 3 DAI (Figs. 1 and 2), but digestion of the basil host cell wall was not conclusively observed or associated with these structures. Adhesion to surfaces is common in fungi and oomycetes (Jones, 1994). Basu et al. (2016) observed “papillae” on hyphae of *R. solani*, fine structures that attach hyphae to host surfaces. Additional studies are needed to determine if the fine structures we observed associated with sporangia play either a role in sporangia attachment or penetration of basil leaves by *P. belbahrii*.

Sporangia germinated at 3 DAI and sporangiophores bearing sporangia were observed by 7 DAI when plants were grown under conditions of near continuous free moisture after inoculation and under a photoperiod of 13 h light and 11 h darkness day^−1^. This is in agreement with earlier studies that reported that leaf wetness for at least 24 h and darkness for at least 7.5 h was needed to achieve infection and dense post-infection sporulation by the basil downy mildew pathogen (Cohen et al., 2013b; Djalali Farahani-Kofoet et al., 2012; Garibaldi et al., 2007). Our results for sporangia germination and sporangiophore production after infection may differ for basil grown under field conditions of varying temperatures and lengths of continuous moisture. Reduced levels of moisture can prevent infection and sporulation (Cohen et al., 2017). Longer periods of free moisture can support maximal sporulation at lower temperatures while shorter periods of wetness can support maximal sporulation at more elevated temperatures for other downy mildew inciting oomycetes (Hyre and Ettinger, 1969; Rotem and Cohen, 1970; Rotem et al., 1976). There are multiple examples of obligate pathogenic downy mildews that can reproduce sexually to produce oospores (Cohen and Sherman, 1977; McMeekin, 1960) including *Peronospora belbahrii* (Elad et al., 2016). However, no oospores were observed under the conditions of the present study, likely due do the inoculum used in our study not consisting of two compatible mating types.

Like other downy mildew pathogens, sporulation of *P. belbahrii* is reported to occur on the abaxial side of infected leaves with dark purplish-brown sporangia being produced during favorable weather conditions (Wyenandt et al., 2015). We observed that sporangia were produced on both sides of leaves in these experiments, especially on young leaves at 10 DAI (Fig. 7). Sporangiophores did, however, tend to be produced first on the underside of younger leaves followed by production on the adaxial surface. Extended conditions of high relative humidity would prevail in greenhouse operations with limited air movement and where basil plant canopies are dense. Under such ideal conditions for infection in the field, sporulation on the top of leaves would likely enhance the spread of the pathogen due to the direct exposure of these sporangia to the dislodging effects of water splash or the movement of plants during production operations.

These SEM results indicate that basil downy mildew disease management through random scouting of fields for disease symptoms is likely an unreliable method. Visible detection of sporangiophore emergence from leaves is symptomatic of advanced disease progression. We recommend development of diagnostic molecular markers of *P. belbahrii* sporangia to enable field based pathogen detection. Portable PCR equipment has been utilized to detect fungal and viral pathogens on important plant commodities (Babu et al., 2017; DeShields et al., 2018).

While recent studies have greatly increased our knowledge of basil downy mildew, considerable knowledge gaps regarding the epidemiology, global population genetics, and control of this disease require additional research in order to further our understanding of how to effectively control *P. belbahrii*.

## Declarations

### Author contribution statement

Guirong Zhang: Conceived and designed the experiments; Performed the experiments; Analyzed and interpreted the data; Wrote the paper.

Arthur Thompson: Performed the experiments; Analyzed and interpreted the data.

David Schisler: Conceived and designed the experiments; Analyzed and interpreted the data; Wrote the paper.

Eric T. Johnson: Analyzed and interpreted the data; Wrote the paper.

### Funding statement

This study was supported by the U.S. Department of Agriculture, Agricultural Research Service.

### Competing interest statement

The authors declare no conflict of interest.

### Additional information

No additional information is available for this paper.
